# Light Field Image Super-Resolution Using Deep Residual Networks on Lenslet Images

**DOI:** 10.3390/s23042018

**Published:** 2023-02-10

**Authors:** Ahmed Salem, Hatem Ibrahem, Hyun-Soo Kang

**Affiliations:** 1 School of Information and Communication Engineering, College of Electrical and Computer Engineering, Chungbuk National University, Cheongju 28644, Republic of Korea; 2 Electrical Engineering Department, Faculty of Engineering, Assiut University, Assiut 71515, Egypt

**Keywords:** light field, image super-resolution, Lenslet images, convolutional neural network

## Abstract

Due to its widespread usage in many applications, numerous deep learning algorithms have been proposed to overcome Light Field’s trade-off (LF). The sensor’s low resolution limits angular and spatial resolution, which causes this trade-off. The proposed method should be able to model the non-local properties of the 4D LF data fully to mitigate this problem. Therefore, this paper proposes a different approach to increase spatial and angular information interaction for LF image super-resolution (SR). We achieved this by processing the LF Sub-Aperture Images (SAI) independently to extract the spatial information and the LF Macro-Pixel Image (MPI) to extract the angular information. The MPI or Lenslet LF image is characterized by its ability to integrate more complementary information between different viewpoints (SAIs). In particular, we extract initial features and then process MAI and SAIs alternately to incorporate angular and spatial information. Finally, the interacted features are added to the initial extracted features to reconstruct the final output. We trained the proposed network to minimize the sum of absolute errors between low-resolution (LR) input and high-resolution (HR) output images. Experimental results prove the high performance of our proposed method over the state-of-the-art methods on LFSR for small baseline LF images.

## 1. Introduction

Light field (LF) captures the intensity and direction of light rays reflected by objects in three-dimensional surroundings. Unlike conventional imaging, which captures the 2D projection of light rays, LF imaging collects data with many dimensions [1]. This abundance of visual information in LF pictures, in addition to their immersive description of the real world, may help several image processing and computer vision tasks, such as depth estimation [2,3], de-occlusion [4,5], salient object detection [6,7], and image post-refocus [8].

Nonetheless, obtaining LF data using plenoptic cameras, such as Raytrix [9], compromises spatial and angular resolutions. Due to a restricted sensor resolution, a plenoptic camera must reduce the spatial resolution of each view to collect more images at a higher angular sampling rate or conversely. Improving LF images’ resolutions is vital, as low-resolution images diminish the performance of low-frequency vision applications. In this paper, we study the LF super-resolution (LFSR) problem.

LFSR methods anticipate subpixel information using the difference between adjacent views [10,11,12,13]. Where these adjacent view images are closely connected in LF, sub-pixel information in each view image may be calculated by leveraging this cross-view correlation, allowing for its super-resolution (SR) reconstruction.

Several deep learning methods with diverse network topologies [14,15,16,17,18,19,20,21,22,23] were recently proposed to accomplish LFSR with enormous LF datasets [24,25,26,27,28]. These methods provide various learning-based SR methods using cross-view correlation through convolutional neural networks (CNN) and transformer-based networks. Although LFSR performance has been steadily improved via careful network design, most present LFSR algorithms underutilize the rich angular information, resulting in performance deterioration, particularly in complicated scenarios. For example, in [19,23], they only considered the spatial and angular information to model the non-local properties of the 4D LF. These methods have been improved upon in [21], by extracting horizontal and vertical epipolar information and spatial and angular information to improve the quality of the resulting images. We argue that Lenslet LF images can provide epipolar and angular information more compactly, allowing the network to model the relationship better and provide more pleasing results [29].

Therefore, this paper proposes a learning-based approach to obtain spatial SR using Lenslet LF images. The Lenslet image is formed by mapping the 4D images into one image using a Periodic Shuffling Operator (PS). We first extract features from input LF images independently. Then, we map the extracted 4D features into Lenslet 2D features. These features are then processed using a sequence of residual groups (RGs) to enhance the spatial resolution and restore some missing details. However, using Lenslet LF images has a blurring effect on the images, which increases with the LF images’ disparity value. Therefore, we process the Lenslet image and sub-aperture images alternately to incorporate angular and spatial information and mitigate the blur effect caused by processing the Lenslet image only. Finally, the final features are aggregated to reconstruct the output image. The quality of the super-resolved images is inversely proportional to the maximum disparity of the scene. In the case of a large disparity, the result is modest and increases inversely with the disparity. In the case of LF with a small disparity, our model comes first in LFSR quality compared with other SR methods.

We conducted several experiments to show our model’s performance in LF super-resolution. The main contributions of our paper are as follows:

- We propose a different paradigm to increase the spatial-angular interaction by processing the Lenslet image and sub-aperture images to incorporate more information for LFSR.
- We propose a CNN-based network to work for LFSR using Lenslet Images with superior performance over the state-of-the-art methods in the case of small-baseline LFSR.
- The remainder of the paper is structured as follows: Section 2 briefly examines the related work. In Section 3, we present our technique for LFSR. Section 4 introduces the conducted experiments to compare our work with the state-of-the-art and discusses the meaning of the obtained results. Finally, Section 5 brings this paper to a close and presents future work to improve the proposed work.

## 2. Related Work

The objective of LF spatial SR, also known as LF image SR, is to produce high-resolution (HR) LF pictures from low-resolution (LR) inputs. Applying independent single image SR (SISR) algorithms to each sub-aperture picture is a basic way of achieving LF spatial SR (SAI). However, straight SISR for LF spatial SR cannot yield adequate results due to the absence of correlation between distinct viewpoints. Therefore, state-of-the-art methods try to fully model the non-local properties of the 4D LF data by using the information inside every single view (i.e., spatial information) and between multiple views (i.e., angular information) to perform well in LFSR. Since using CNNs in the field of LFSR, their utilization has grown exponentially, and the reconstruction performance has improved continually. Zhang et al. [14] presented a residual network for LFSR. The images in four directions are first stacked and given to separate branches to extract sub-pixel correlations. Then the information from these branches is combined for the final reconstruction. Because just a few side views may be employed, the performance of side views will suffer when compared to the performance of the center view, resulting in undesired inconsistencies in the reconstructed LF pictures. The performance of their later work [15] was increased considerably by using 3D convolutions applied to view image stacks of diverse angle orientations. Jin et al. [16] utilized an all-to-one technique for LFSR and used structural consistency regularization to retain the parallax structure. Yeung et al. [17] proposed to shuffle LF spatial-angular features alternately at a single forward pass using separable convolutions. Wang et al. [18] used deformable convolution on LF images to overcome the disparity problem for LFSR. Wang et al. [19] presented an interactive network (LF-InterNet). In particular, spatial and angular features are extracted and repeatedly interact to extract complementary information step by step. Then, each view image is super-resolved by fusing the interacting features. A network with two parallel branches was suggested by Liu et al. [20]. The top one collects global interview data. The bottom one separately projects each view to deep representations and then models the correlations between all intra-view characteristics using a multi-view context block. Wang et al. [21] designed a disentangling approach by dividing LF into several subspaces. They extracted features using three feature extractors (spatial, angular, horizontal, and vertical epipolar information). The network’s convolution layers only need to analyze information in a single subspace, facilitating LF representation learning. Different from CNN and inspired by recent achievements in Transformers [30], Wang et al. [22] developed a detail-preserving Transformer (DPT) to recover the features of light field (LF) pictures by using gradient maps of light field to direct sequence learning. However, the frameworks of these techniques are all-inclusive models whose supplementary information is not effectively employed for performance enhancement. Later, Liang et al. [23] suggested a Transformer-based LF image SR network in which a spatial Transformer and an angular Transformer were built to simulate, respectively, long-range spatial interdependence and angular correlation.

## 3. Our Approach

### 3.1. Problem Formulation

**Figure 1:**
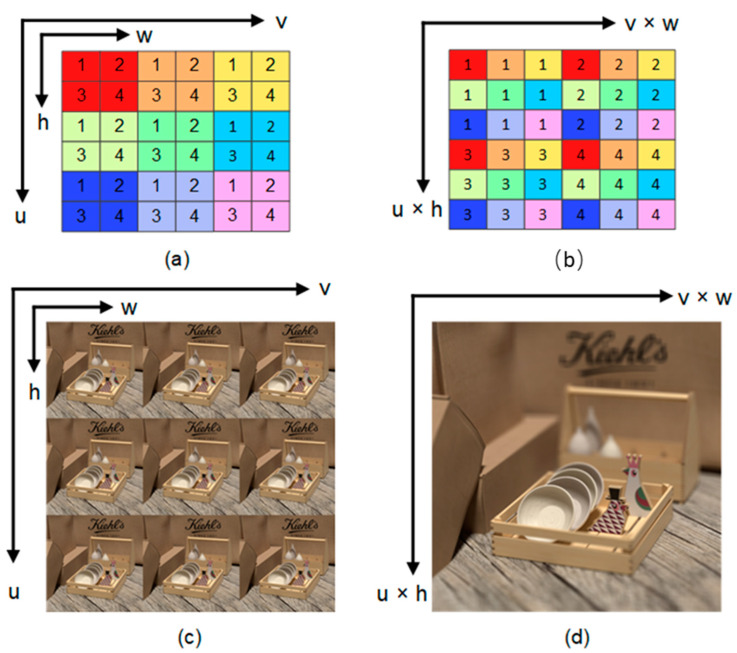
4D vs. Lenslet LF representation. (**a**) 4D representation: 3 × 3 LF images where each image has four pixels, and each image is represented by a different color, (**b**) The mapping from the 4D LF of size (u, v, h, w) into the Lenslet image of size (u × h, v × w) using the shuffling operator, (**c**) a real example of LF scene represented by 3 × 3 images, (**d**) the Lenslet LF image of the 3 × 3 images in (**c**).

In our approach, the LF is formulated as a 2D array of Sub-Aperture Images (SAI), as shown in Figure 1c, and given by $L\in {\;R}^{u\;\times \;v\;\times \;h\;\times \;w}$, with (h, w) and (u, v) spatial and angular resolutions. Therefore, given a low-resolution LF input as $L_{\mathrm{LR}}\in {\;R}^{u\;\times \;v\;\times \;h\;\times \;w}$, we aim to reconstruct its high-resolution counterpart $L_{\mathrm{HR}}\in {\;R}^{u\;\times \;v\;\times \;\alpha h\;\times \;\alpha w}$, by enhancing the spatial resolution, where α represents the super-resolution factor. Following recent approaches [14,15,16,17,18,19,20,21,22,23], we set ∝=2,4 and assume that SAIs are distributed in a square array, i.e., u = v = A, where A represents the vertical or horizontal angular resolution. Before feeding $L_{\mathrm{LR}}$ to the network, we up-sample the input LF epipolar plane images (EPIs) to the desired output size with α utilizing the Bicubic interpolation. Finally, we arrange the input from the 4D representation into the 3D representation $L_{\mathrm{LR}}\in {\;R}^{\mathrm{uv}\;\times \;h\;\times \;w}$.

### 3.2. Features Extractors

**Figure 2:**
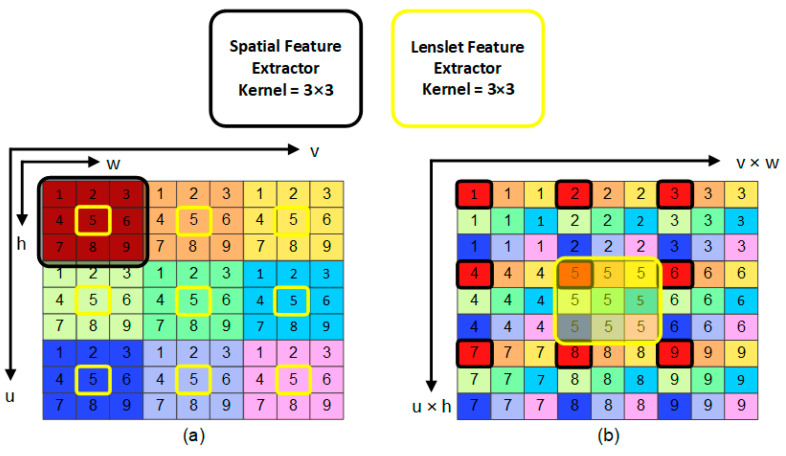
An illustration of the spatial and Lenslet feature extractors. Here, we have 3 × 3 LF images with nine pixels in each view image and a different color. The spatial feature extractor is shown in black color, while the Lenslet extractor is shown in yellow. We utilize a convolution filter for both extractors with a kernel of size 3 × 3 and a stride of 1. The spatial extractor is applied to each view image independently, as shown in (**a**), while the Lenslet extractor is applied to the Lenslet image, as shown in (**b**).

We aim to extract spatial information along with horizontal epipolar, vertical epipolar, and angular information to model the non-local properties of the 4D LF fully. We utilize a convolution filter with a kernel of size 3 × 3 as a spatial feature extractor, as shown in black in Figure 2. This filter is applied to SAI separately. To extract horizontal epipolar, vertical epipolar, and angular information, we utilize another convolution filter with a kernel of size 3 × 3 as a Lenslet feature extractor, as shown in yellow in Figure 2. However, we apply this filter to the Lenslet image. The idea behind operating on the Lenslet image is that rows of the Lenslet image represent horizontal epipolar lines, columns of the Lenslet image represent vertical epipolar lines, and pixels in the Lenslet image represent the angular information. Therefore, one of the key benefits of dealing with a Lenslet image is extracting a large amount of information with a single convolution filter.

### 3.3. Network Overview

**Figure 3:**
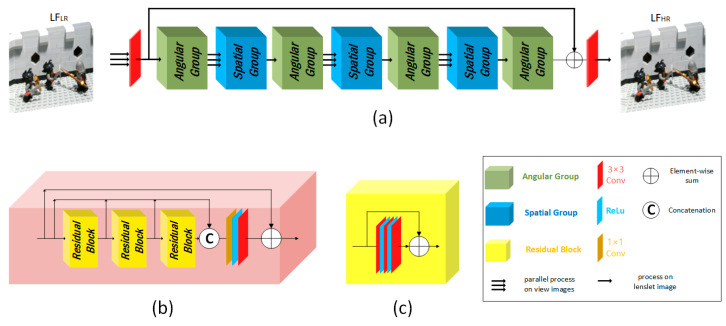
(**a**) The overall design of our network for LFSR. (**b**) The residual group (RG) design is used as an angular and spatial group in (**a**). (**c**) Residual block (RB) design.

In our method, we process the Lenslet LF similar to the method proposed in [29] for LF angular super-resolution. At the same time, the proposed architecture is designed similarly to the deep residual channel attention networks [31]. Figure 3 depicts the overall design of our network. The proposed network consists of three cascaded stages: initial feature extraction, convolutional neural network (CNN)-based super-resolution network, and final image reconstruction, as shown in Figure 3a. The first and last stages of the network consist of a single 3 × 3 convolution layer, and the middle consists of a long skip connection with cascaded residual angular and residual spatial groups (RG). The angular and spatial groups share the same structure, as shown in Figure 3b. The input LF is processed differently by different network components. For example, the initial feature extractor and spatial groups process each 2D view image ${\mathrm{VI}}_{\mathrm{LR}}\in {\;R}^{h\;\times \;w}$ independently. While angular groups and the final reconstruction stage process the 2D Lenslet image $L_{\mathrm{LR}}\in {\;R}^{\mathrm{uh}\;\times \;\mathrm{vw}}$.

**Figure 4:**
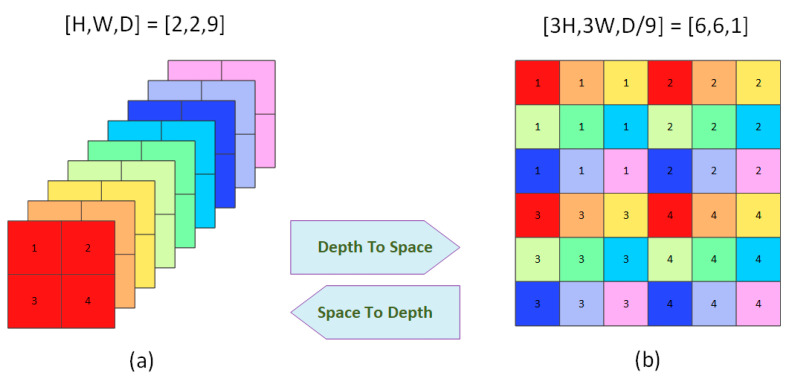
Periodic shuffling (PS) operation (Depth-to-Space and Space-to-Depth). (**a**) A sequence of view images with size (H, W, D). (**b**) Lenslet Image with size (3H, 3W, D/9) contains all the view images in a different order. The mapping from (**a**) to (**b**) is called depth-to-space, while from (**b**) to (**a**) is called space-to-depth.

The mapping between the 3D LF $\in {\;R}^{\mathrm{uv}\;\times \;h\;\times \;w}$ and the 2D Lenslet LF $\in {\;R}^{\mathrm{uh}\;\times \;\mathrm{vw}}$ is done using a periodic shuffling operator (PS) [32,33], as shown in Figure 4. In Figure 3a, the three arrows before each block indicate that this block processes each view image independently, while other blocks process the Lenslet image. Initial features are extracted in the first stage of the network to be fed to the main part of the network for processing, where features are extracted from each view image independently and then rearranged to the Lenslet image using a PS. The main part of the network consists of a long skip connection with cascaded angular and spatial RGs. Each RG consists of three residual blocks (RB) in our implementation. Each RB has three cascaded convolution layers with a ReLU in between with a skip connection.

### 3.4. Loss Function and Training Details

We trained our network only on the luminance component while we upsample the chrominance components using the Bicubic interpolation. We trained our network to learn a mapping from the Low-Resolution (LR) LF image ${\mathrm{LF}}_{\mathrm{LR}}$ to the High-Resolution (HR) LF image ${\mathrm{LF}}_{\mathrm{HR}}$. We can write the problem can as follows:

(1) $${{\mathrm{LF}}^{\prime }}_{\mathrm{HR}}=f\left({\mathrm{LF}}_{\mathrm{LR}},\;\theta \right)$$

where f(.) is the function mapping from the LR image to the HR image, and θ is the network parameters to be learned while training.

We trained the proposed network to reduce the L_1_ distance. L_1_ loss is defined as follows, given a training set with N pairs of LR input and HR output images:

(2) $$L_{1}\left(\theta \right)=\frac{1}{N}\sum_{i=1}^{N}{\left|{\mathrm{LF}}_{\mathrm{HR}}^{i}-f\left({\mathrm{LF}}_{\mathrm{LR}}^{i}\right)\right|}_{1}$$

Following recent approaches [14,15,16,17,18,19,20,21,22,23], we used 144 LF images for training and 23 for testing from publicly available synthetic and real-world datasets [24,25,26,27,28], as shown in Table 1. The original angular resolution of these datasets is 9 × 9, while we used the middle 5 × 5 views for training and testing. These datasets are divided into three categories based on their disparity value. For example, small-disparity LF includes EPFL and INRIA, medium-disparity LF includes HCInew and HCIold, and large-disparity LF includes STFgantry. The HCInew and HCIold are Synthetic, while other datasets are real-world datasets captured by the Lytro Illum camera.

We extract patches of size 32 × 32 with a stride of one from input and ground-truth images to prepare the training dataset. Our model was trained by ADAM optimizer [34] with β1 = 0.9, β2 = 0.999, and ǫ = 10^−8^. The initial learning rate is set to 2 × 10^−4^ and then decreases exponentially by 0.1 every 80 epochs. Our model was trained for 85 epochs in Tensorflow [35] with NVIDIA TITAN RTX GPU.

## 4. Experiments and Discussion

### 4.1. Comparison with the State-of-the-Art Methods

To demonstrate the performance of our model in the LFSR task, we compare it with state-of-the-art single image SR (SISR) methods, including VDSR [36], EDSR [37], RCAN [31], and LFSR methods, including resLF [14], MEG-Net [15], LF-ATO [16], LFSSR [17], LF-InterNet [19], LF-DFnet [18], LF-IINet [20], DPT [22], LFT [23], DistgSSR [21] with all models retrained with the same datasets.

#### 4.1.1. Quantitative Comparison

Average PSNR and SSIM are used for the luminance images over all the output views to measure the super-resolution quality. We present numerical results in terms of (PSNR/SSIM) in Table 2 and for 2× and 4× LFSR, respectively. The best results are shown in red, and the second-best results are in blue. The quality of the super-resolved images is inversely proportional to the maximum disparity of the scene. In the case of a large disparity, the result is modest and increases inversely with the disparity. In the case of LF with a small disparity, our model comes first compared with other SR methods and achieves competitive PSNR and SSIM. For example, 0.96 dB and 0.23 dB are higher than the LFT method [23] on the EPFL dataset for 2× and 4×, respectively. Moreover, 1.85 dB and 0.88 dB are higher than the LFT method [23] on the INRIA dataset for 2× and 4×, respectively.

#### 4.1.2. Qualitative Comparison

**Figure 5:**
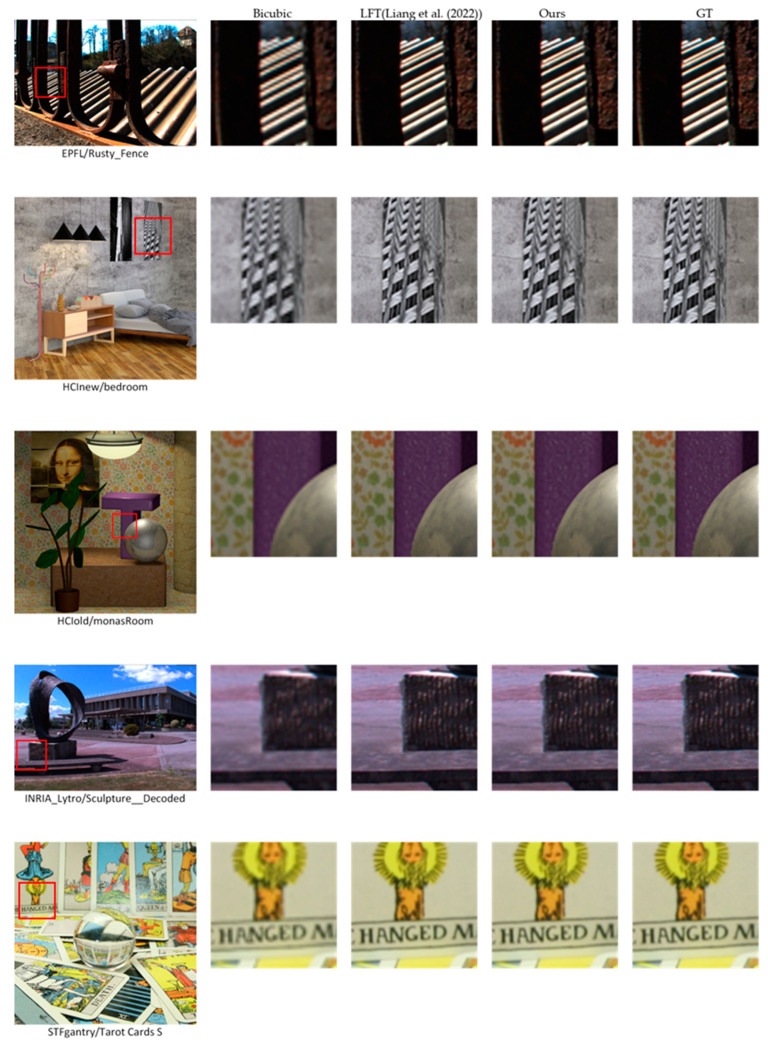
Visual comparison for different 2× LFSR methods. We chose one scene from each dataset for comparison. In addition, a close-up of image portions in red boxes is provided for Bicubic, LFT [23], our method, and ground truth, respectively.

We compare our results for 2× LFSR visually with one of the state-of-the-art methods [23], as shown in Figure 5. However, it isn’t easy to differentiate between output images as they are very similar, so we urge the reader to check Table 2 and Table 3 for more details. We attribute the significant improvement in the case of small-disparity LF to Lenslet images, which permits the network to accurately understand and simulate the linking between different views of the same scene, recover more texture information, and improve the quality.

#### 4.1.3. Model Efficiency

We compare our proposed model to several competitive methods in terms of the number of parameters, average PSNR, and average SSIM scores. As presented in Table 4, our model achieves the highest PSNR score and second-highest SSIM score for 2× LFSR. For 4× LFSR, our model achieves the second-highest PSNR score and second SSIM score. Except for LFT [23], a transformer-based model, our model is considered the best regarding the number of parameters and average PSNR and SSIM.

### 4.2. Ablation Study

In this subsection, we validate the effectiveness of two parameters on the final results, including our approach to using Lenslet extractors along with spatial extractors and the patch size used to train the network.

#### 4.2.1. Feature Extractors

As shown in Table 5, we examine three alternatives to highlight the impact of the model’s various components. First, we train the proposed model utilizing the spatial feature extractor only and then utilizing the Lenslet feature extractor only. Finally, we train the proposed model using spatial and Lenslet feature extractors. It is clear from the results that the spatial extractor or Lenslet extractor alone cannot super-resolve LF images with high quality. It is also shown that even though the Lenslet extractor cannot super-resolve high-quality LF images, it may give better results when compared to the spatial extractor alone as it can extract angular information along with the epipolar information.

However, when comparing the model’s efficiency trained using only Lenslet extractor on the small-disparity LF, including EPFL and INRIA, this model performs better than the state-of-the-art methods, which validates our point that Lenslet images can provide a huge amount of information regarding the relationship between different view images.

#### 4.2.2. Patch Size

As shown in Table 6 and Table 7, we examine the effect of patch size on the model using three different sizes 16 × 16, 32 × 32, and 64 × 64. When training for 2× LFSR, the 16 × 16 shows a slightly better performance than the 32 × 32. However, when training for 4× LFSR, the 32 × 32 achieves the best performance. The patch size affects the model trained for 4× LFSR more, especially with the degradation of the input image more than the 2× model.

## 5. Conclusions and Future Work

In this paper, we proposed a residual convolutional network for LFSR. To effectively explore the non-local property of 4D LF, we adopted the Lenslet LF representation. The Lenslet representation is compact and can provide abundant information. The Lenslet rows represent horizontal epipolar lines, columns represent vertical epipolar lines, and pixels in the Lenslet image represent angular information. Therefore, we alternately process the Lenslet image and view images to incorporate angular and spatial information. We used five datasets for training and testing, and our proposed model achieves the highest average PSNR and the second-highest average PSNR on 2× and 4× LFSR, respectively. In addition, experimental results show that the spatial or Lenslet extractor alone cannot super-resolve LF pictures well. The Lenslet extractor can extract epipolar and angular information; therefore, even though it cannot super-resolve high-quality LF pictures, it may produce better results than the spatial extractor alone. Furthermore, using three different patch sizes, 16 × 16, 32 × 32, and 64 × 64 to train the model, we found that 32 × 32 achieves the best performance on 2× and 4× LFSR.

However, the Lenslet image can provide much information for adjacent views at the same location, but it fails to provide the same amount of information for scenes with large disparity. Therefore, our model shows high performance with images with small disparity and poor performance with images with high disparity because performance is inversely proportional to the maximum disparity in the scene. In the future, we plan to:

- Shear LF images into different disparity levels; after shearing, the disparity value will become smaller, then our network can extract better features, as proposed in [38].
- Use a parallax-attention module (PAM) as a final stage, where PAM was designed to capture a global correspondence in stereo images super-resolution [39].
- Adopt a transformer-based architecture, where transformers can understand the local and global features that benefit images with large disparities, such as the vision [30] and swing transformers [40].

## Figures and Tables

**Table 1:** LF datasets used for training and testing.

Dataset	Training	Testing	Disparity	Data Type
HCInew [24]	20	4	[−4, 4]	Synthetic
HCIold [26]	10	2	[−3, 3]	Synthetic
EPFL [25]	70	10	[−1, 1]	Real-world
INRIA [27]	35	5	[−1, 1]	Real-world
STFgantry [28]	9	2	[−7, 7]	Real-world

**Table 2:** Numerical comparison for different 2× LFSR methods. The best results are in red, and the second-best in blue.

Dataset	EPFL	HCInew	HCIold	INRIA	STFgantry	Average
Bicubic	29.74/0.941	31.89/0.939	37.69/0.979	31.33/0.959	31.06/0.954	32.34/0.954
VDSR [36]	32.50/0.960	34.37/0.956	40.61/0.987	34.43/0.974	35.54/0.979	35.49/0.971
EDSR [37]	33.09/0.963	34.83/0.959	41.01/0.988	34.97/0.977	36.29/0.982	36.04/0.974
RCAN [31]	33.16/0.964	34.98/0.960	41.05/0.988	35.01/0.977	36.33/0.983	36.11/0.974
resLF [14]	33.62/0.971	36.69/0.974	43.42/0.993	35.39/0.981	38.36/0.990	37.50/0.982
LFSSR [17]	33.68/0.974	36.81/0.975	43.81/0.994	35.28/0.983	37.95/0.990	37.51/0.983
MEG-Net [15]	34.30/0.977	37.42/0.978	44.08/0.994	36.09/0.985	38.77/0.991	38.13/0.985
LF-ATO [16]	34.27/0.976	37.24/0.977	44.20/0.994	36.15/0.984	39.64/0.993	38.30/0.985
LF-InterNet [19]	34.14/0.972	37.28/0.977	44.45/0.995	35.80/0.985	38.72/0.992	38.08/0.984
LF-DFnet [18]	34.44/0.977	37.44/0.979	44.23/0.994	36.36/0.984	39.61/0.993	38.42/0.985
LF-IINet [20]	34.68/0.977	37.74/0.979	44.84 /0.995	36.57/0.985	39.86/0.994	38.74/0.986
DPT [22]	34.48/0.976	37.35/0.977	44.31/0.994	36.40/0.984	39.52/0.993	38.41/0.984
LFT [23]	34.80/0.978	37.84/0.979	44.52/0.995	36.59/0.986	40.51/0.994	38.85/0.986
DistgSSR [21]	34.80/ 0.979	37.95/0.980	44.92/0.995	36.58/0.986	40.37/ 0.994	38.92/0.987
Ours	35.76/0.979	37.49/0.979	44.50/0.994	38.44/0.986	39.16/0.993	39.05 /0.986

**Table 3:** Numerical comparison for different 4× LFSR methods. The best results are in red, and the second-best in blue.

Dataset	EPFL	HCInew	HCIold	INRIA	STFgantry	Average
Bicubic	25.14/0.833	27.61/0.853	32.42/0.931	26.82/0.886	25.93/0.847	27.58/0.870
VDSR [36]	27.25/0.878	29.31/0.883	34.81/0.952	29.19/0.921	28.51/0.901	29.81/0.907
EDSR [37]	27.84/0.886	29.60/0.887	35.18/0.954	29.66/0.926	28.70/0.908	30.20/0.912
RCAN [31]	27.88/0.886	29.63/0.888	35.20/0.954	29.76/0.927	28.90/0.911	30.27/0.913
resLF [14]	28.27/0.904	30.73/0.911	36.71/0.968	30.34/0.941	30.19/0.937	31.25/0.932
LFSSR [17]	28.27/0.908	30.72/0.912	36.70/0.969	30.31/0.945	30.15/0.939	31.23/0.935
MEG-Net [15]	28.74/0.916	31.10/0.918	37.28/0.972	30.66/0.949	30.77/0.945	31.71/0.940
LF-ATO [16]	28.52/0.912	30.88/0.914	37.00/0.970	30.71/0.949	30.61/0.943	31.54/0.938
LF-InterNet [19]	28.67/0.914	30.98/0.917	37.11/0.972	30.64/0.949	30.53/0.943	31.59/0.939
LF-DFnet [18]	28.77/0.917	31.23/0.920	37.32/0.972	30.83/0.950	31.15/0.949	31.86/0.942
LF-IINet [20]	29.11/0.920	31.36/0.921	37.62/0.974	31.08/0.952	31.21/0.950	32.08/0.943
DPT [22]	28.93/0.917	31.19/0.919	37.39/0.972	30.96/0.950	31.14/0.949	31.92/0.941
LFT [23]	29.25/0.921	31.46/0.922	37.63/0.974	31.20/0.952	31.86/0.955	32.28/0.945
DistgSSR [21]	28.98/0.919	31.38/0.922	37.55/0.973	30.99/0.952	31.63/0.953	32.11/0.944
Ours	29.48/0.922	31.01/0.921	37.17/0.971	32.08/0.953	30.83/0.951	32.26/0.944

**Table 4:** Comparison of the number of model parameters and average PSNR and SSIM for 2× and 4× LFSR. The best results are in red, and the second-best in blue.

Dataset	2×			4×		
	#Param.	PSNR	SSIM	#Param.	PSNR	SSIM
EDSR [37]	38.6 M	36.04	0.974	38.9 M	30.20	0.912
RCAN [31]	15.3 M	36.11	0.974	15.4 M	30.27	0.913
resLF [14]	6.35 M	37.50	0.982	6.79 M	31.25	0.932
LFSSR [17]	0.81 M	37.51	0.983	1.61 M	31.23	0.935
MEG-Net [15]	1.69 M	38.13	0.985	1.77 M	31.71	0.940
LF-ATO [16]	1.51 M	38.30	0.985	1.66 M	31.54	0.938
LF-InterNet [19]	4.80 M	38.08	0.984	5.23 M	31.59	0.939
LF-DFnet [18]	3.94 M	38.42	0.985	3.99 M	31.86	0.942
LF-IINet [20]	4.84 M	38.74	0.986	4.89 M	32.08	0.943
DPT [22]	3.73 M	38.41	0.984	3.78 M	31.92	0.941
LFT [23]	1.11 M	38.85	0.986	1.16 M	32.28	0.945
DistgSSR [21]	3.53 M	38.92	0.987	3.58 M	32.11	0.944
Ours	3.21 M	39.05	0.986	3.21 M	32.26	0.944

**Table 5:** Numerical comparison for variants of our proposed network using different feature extractors for 2× LFSR. The best results are in red, and the second-best in blue.

Dataset	EPFL	HCInew	HCIold	INRIA	STFgantry	Average
Spatial	33.35/0.964	34.55/0.960	40.68/0.987	35.58/0.977	35.99/0.983	36.03/0.974
Lenslet	35.06/0.976	36.40/0.975	43.35/0.993	37.63/0.985	36.95/0.989	37.88/0.984
Both	35.76/0.979	37.49/0.979	44.50/0.994	38.44/0.986	39.16/0.993	39.05/0.986

**Table 6:** Numerical comparison for variants of our proposed network trained with different patch sizes for 2× LFSR. The best results are in red, and the second-best in blue.

Dataset	EPFL	HCInew	HCIold	INRIA	STFgantry	Average
16 × 16	35.78/0.980	37.46/0.979	44.34/0.994	38.47/0.987	39.36/0.993	39.08/0.987
32 × 32	35.76/0.979	37.49/0.979	44.50/0.994	38.44/0.986	39.16/0.993	39.05/0.986
64 × 64	35.46/0.977	37.17/0.977	44.14/0.994	38.11/0.985	38.98/0.993	38.77/0.985

**Table 7:** Numerical comparison for variants of our proposed network trained with different patch sizes for 4× LFSR. The best results are in red, and the second-best in blue.

Dataset	EPFL	HCInew	HCIold	INRIA	STFgantry	Average
16 × 16	29.33/0.918	30.63/0.912	36.63/0.967	31.44/0.949	30.21/0.940	31.65/0.937
32 × 32	29.48/0.922	31.01/0.921	37.17/0.971	32.08/0.953	30.83/0.951	32.26/0.944
64 × 64	29.34/0.918	30.80/0.919	36.77/0.969	31.84/0.951	30.51/0.948	31.85/0.941

## Data Availability

The datasets used in this paper are public datasets. We also provide the proposed method’s test and evaluation codes at: https://github.com/ahmeddiefy/LFSR_Lenslet, created and accessed on 19 December 2022.
